# Multimodal integration supports neural processing of grammatical negation

**DOI:** 10.1073/pnas.2605004123

**Published:** 2026-09-16

**Authors:** Hatice Zora, Peter Hagoort, Aslı Özyürek

**Affiliations:** ^a^ Max Planck Institute for Psycholinguistics, Nijmegen 6525 XD, The Netherlands; ^b^ Donders Institute for Brain, Cognition and Behaviour, Radboud University, Nijmegen 6525 HT, The Netherlands

**Keywords:** multimodality, prosody, gesture, negation, EEG

## Abstract

Negation is a fundamental aspect of human cognition and grammar, expressed across different modalities. Yet, little is known about multimodal processing of negation, especially grammatical negation (e.g., “I did *not* sleep”), and the contribution of gestures and prosodic markers to its neural processing remains unexplored. This study investigates the neurocognition of multimodal negation in Turkish, a verb-final language where standard verbal negation is expressed morphologically with a postverbal suffix (*-mA*). Notably, this negation suffix shifts stress onto the verb stem, deviating from the default final-stress pattern of Turkish. In Turkish, standard verbal negation is also frequently accompanied by conventional negation gestures (backward hand flips), occurring on the verb stem preceding the negation suffix. Since both gestures and prosodic markers precede the negation suffix, we examined whether and how their presence affects the postverbal negation processing using electroencephalography. Gestures elicited an N100 effect driven by their inherent perceptual salience. Gestures also reduced the N400 amplitude in anticipation of the postverbal negation suffix, pointing to a facilitative role of negation gestures in negation processing. Prosody likewise strengthened this N400 reduction, but only when gestures were present. These findings demonstrate that gestures—and to a lesser extent prosody—reduce the cognitive effort involved in processing the morphosyntactic marker of verbal negation. Overall, the results provide neurophysiological evidence that multimodal information supports not only semantic processing, as shown before, but also core grammatical operations in language.

## Significance

Contrary to classical unimodal linguistic theories, there is growing consensus supporting the view of language as a multimodal system. The present study provides neurocognitive evidence for this view by examining negation, a core aspect of human cognition and grammar. Using electroencephalography, we investigated how prosody and gestures influence standard verbal negation processing in Turkish, ideal for this purpose. In Turkish, standard verbal negation is expressed with a postverbal morphological marker, whereas gesture and prosodic prominence occur on the preceding verb. Results show that gestures reduce cognitive effort in verbal negation processing, with prosody contributing only alongside gestures. These findings suggest that grammatical negation relies on multimodal integration, consistent with proposals that gestures are part of the grammatical encoding of language.

In recent years increasing evidence has suggested that, in contrast to classical unimodal linguistic theories, language structures and processing are inherently multimodal. In this context, an important example is negation. Negation is a core aspect of human cognition, universally encoded in the grammar of the world’s languages (1–3). While negation in propositional logic simply inverts a statement’s truth value, negation in natural languages is more complex, taking multiple forms and interacting with diverse linguistic functions at the level of lexicon and grammar as well as semantics and pragmatics (2, 4–6).

While initially studied in text and speech, the multimodal nature of negation has been increasingly recognized (7–11). In face-to-face interaction, prosodic and gestural cues co-occur with negative expressions to convey denial, rejection, and corrective speech acts. A specific set of manual and nonmanual expressions has been identified as systematically associated with negation, such as head shakes, palm-down gestures, and eyebrow frowns, often accompanied by characteristic intonational patterns (10). Notably, these cues play a key role in shaping the semantic scope and the pragmatic interpretation of negation. For instance, Prieto et al. (9) show that intonational contours, together with a combination of manual and nonmanual gestures, are crucial for pragmatic inferencing, especially in the interpretation of double negation, aligning well with the relevance-theoretic views where any input to cognitive processing may be relevant at a given moment. Likewise, by examining multimodal negation such as accented negation words, accompanying lateral sweeping hand movements, and facial expressions, Harrison (11) identified an acoustic pattern that appears to be closely linked with the form and organization of gestures.

However, prior work has largely focused on semantic composition at the level of negation words and pragmatic aspects at the level of speech acts, and has not addressed how these features interact at the morphosyntax level, specifically in the grammatical encoding of standard negation, or the neurocognitive mechanisms underlying their processing. This study aims to examine the neural correlates of multimodal negation, focusing on the contributions of prosody and gesture. We will investigate this in Turkish, a language in which both prosody and negation gesture play an important role in marking negation.

Cospeech gestures have been shown to interact with neural processing of language mostly at the semantic level (12) but not at the grammatical level. Furthermore, comparatively little attention has been given to the role of negation gestures in language structure and processing, which are conventionalized and culturally specific (13). The neurocognitive relevance of prosody in language processing is well documented across diverse linguistic functions, including grammatical processing, and languages (14, 15; cf. 16), including Turkish (17, 18). Yet a neurocognitive framework investigating interactions between prosody and gesture supporting negation is lacking.

Typological studies on grammatical negation have mainly examined declarative clauses with verbal predicates, i.e., standard negation (19), which is the focus of this paper. Dahl (1) distinguishes two types: syntactic negation, where the negative marker is a particle or auxiliary (e.g., English particle not: “John is not swimming”) and morphological negation, where it is an affix (e.g., Turkish suffix -mA, the capital letter marking vowel-harmonic alternation): “gel-me-dim” [come-NEG-PAST-1SG] “I did not come.” Negation typically appears early, often before the verb (5), and negation particles follow this pattern across word orders (1, 20). However, morphological negation slightly favors suffixes and is more common in verb-final or free word order languages (1).

**Fig. 1.**
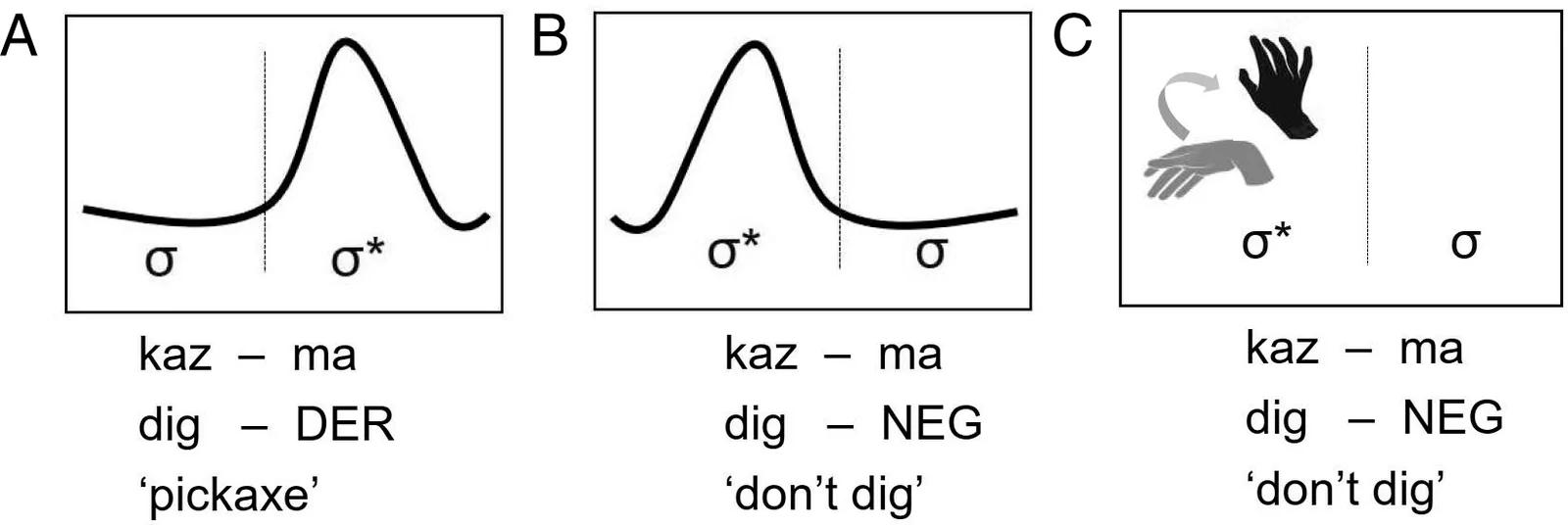
A prosodic and gestural overview of standard negation in Turkish. (*A*) Illustration of final stress, where the stressable derivational (DER) suffix –mA is attached to the verb stem. (*B*) Illustration of nonfinal stress, where the prestressing negative suffix –mA is attached to the verb stem. (*C*) Illustration of a backward hand flip gesture preceding the negation suffix. Sigma (σ) labels each syllable, and asterisks (*) indicate stressed syllables.

Turkish, a verb-final agglutinative language, marks standard negation morphologically with the suffix –mA, which attaches to the verb stem and carries a distinctive prosody. In Turkish, word level prominence (stress hereafter) typically falls on the final syllable regardless of morphological complexity. However, the negation suffix –mA behaves as a prestressing suffix, shifting stress to the syllable directly before it (the verb stem in the present study) (Fig. 1 *A* and *B*; 21). Negation in Turkish is also frequently accompanied by a conventional gesture, namely backward hand flip, which precedes the negation suffix (22; Fig. 1*C*).

Crucially, the backward hand flip—a potentially geographically and culturally specific negation gesture, with analogous forms found in Turkish and Greek Sign Languages (23) and possibly others in the Mediterranean and Middle East regions—functions in a morpheme-like manner to express grammatical negation in Turkish. This sets it apart from other negation strategies such as lateral hand/finger and head movements associated with “never” or “no” and their accompanying multimodal cues. It is also important to distinguish between verbal negation and copular negation. While some languages use the same negation marker (e.g., not in English is used in both verbal negation, as in “John is not swimming” and copular negation, as in “John is not a doctor”), Turkish expresses copular negation with “değil” (e.g., John doktor değil “John is not a doctor”), and -mA is restricted to verbal negation (e.g., “gel-me-dim” “I did not go”).

Cross-linguistically, negators often precede the verb, reflecting Hawkins’s (24) principle of Maximizing Online Processing (MaOP) (4). According to Hawkins, grammars are shaped by the demands of language processing, and at the core of the MaOP principle is the idea that efficiency is enhanced by choosing and organizing linguistic forms in ways that allow the earliest possible access to the syntactic and semantic representations. In Turkish, the negation suffix -mA follows the verb, but preceding prosodic and gestural markers may facilitate processing of negation in line with the MaOP.

Turkish prosodic marking of standard verbal negation appears grammaticalized as many other bound morphemes including enclitics and particles (21) and encoded at the lexical level preceding the morphosyntactic marker rather than being expressed through intonational contour variation, while negation gestures although optional are prevalent in the corpus, appearing in approximately 85% of instances of sentences with standard verbal negation (25). This raises important questions about how prosody and the negation gesture independently or jointly interact with the negation suffix, and consequently influence the grammatical processing of negation. Using electroencephalography (EEG), we investigate this interaction, examining amplitude modulations in event-related potentials (ERPs) that reveal any processing advantage associated with these markers.

## Results

**Fig. 2.**
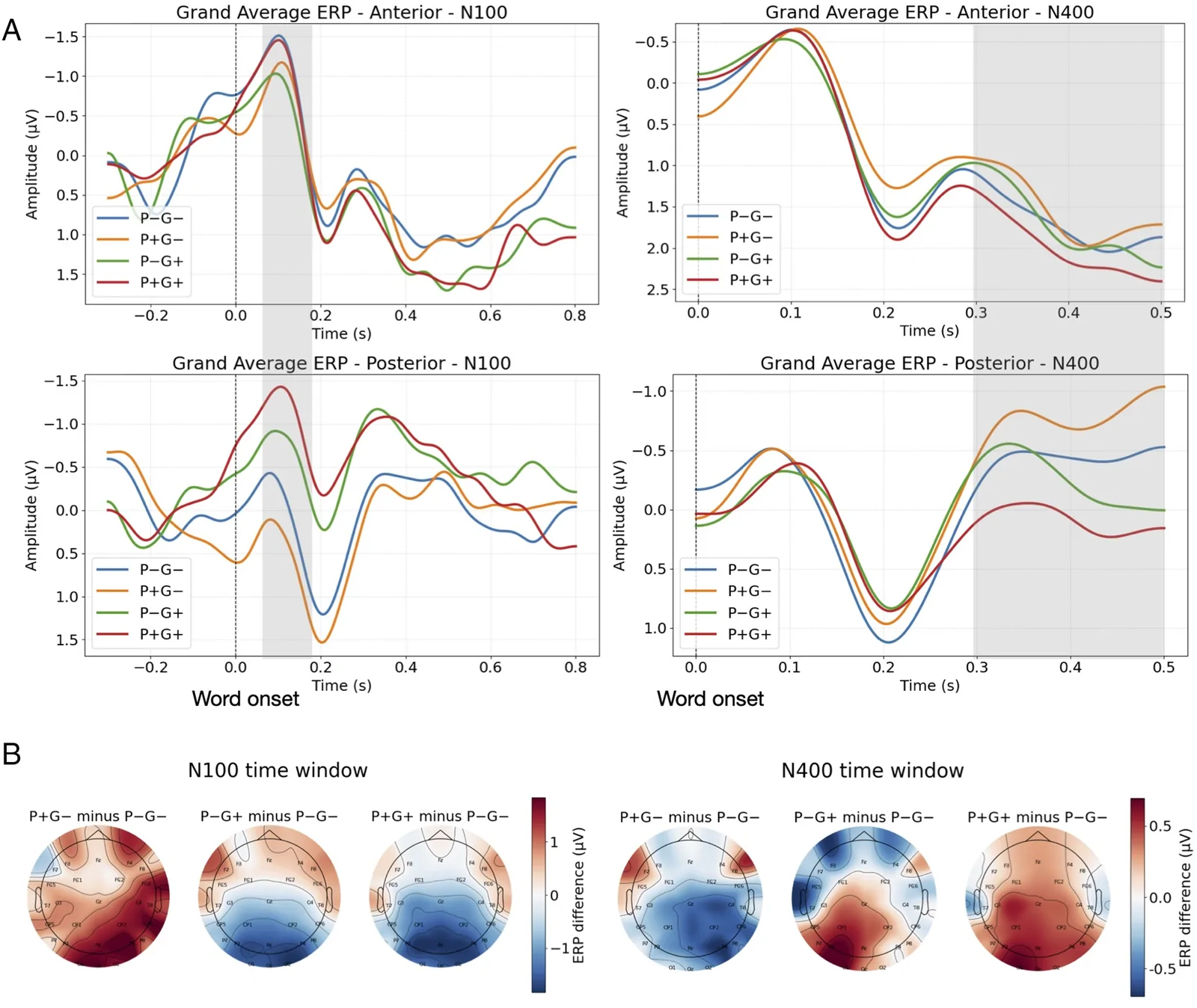
Overview of the EEG results. (*A*) Grand average ERP waveforms elicited by experimental conditions (P+G+: prosody present, gesture present, red line; P+G–: prosody present, gesture absent, orange line; P–G+: prosody absent, gesture present, green line; P–G–: prosody absent, gesture absent, blue line) in the anterior and posterior ROIs in the N100 (50 to 150 ms) time window (*Left*). Grand average ERP waveforms elicited by experimental conditions (P+G+: prosody present, Gesture present, red line; P+G–: prosody present, gesture absent, orange line; P–G+: prosody absent, gesture present, green line; P–G–: prosody absent, gesture absent, blue line) in the anterior and posterior ROIs in the N400 (300 to 500 ms) time window with adjusted baseline correction (*Right*). Amplitude is reported in microvolts (µV) and time in seconds (s). Negativity is plotted upward. Shaded areas indicate the relevant time windows. Word onset represents verb onset. (*B*) Topographic distributions for the experimental conditions (P+G+, P−G+, P+G−), relative to the baseline condition (P−G−), are presented for the two time windows: N100 (50 to 150 ms) and N400 (300 to 500 ms). Amplitude is reported in microvolts (µV).

**Fig. 3.**
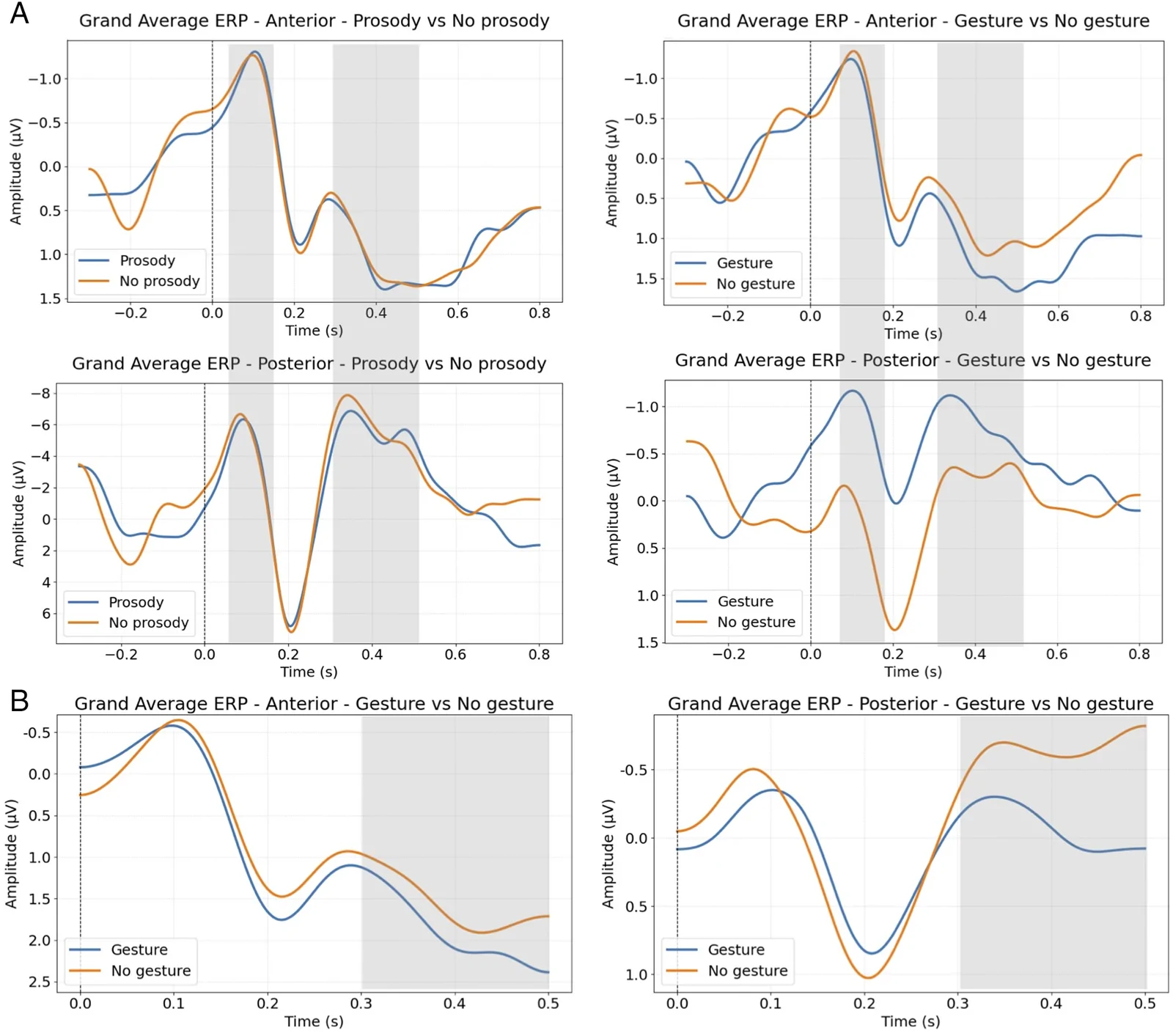
Overview of the collapsed EEG results. (*A*) Grand average ERP waveforms elicited by the prosody condition (P+G− and P+G+ collapsed) compared with the no prosody condition (P−G− and P−G+ collapsed) (*Left*) as well as the gesture condition (P−G+ and P+G+ collapsed) compared with the no gesture condition (P−G− and P+G− collapsed) (*Right*) in the anterior and posterior ROIs in the N100 (50 to 150 ms) time window and the N400 (300 to 500 ms) time window (without adjusted baseline correction). (*B*) Grand average ERP waveforms elicited by the gesture condition (P−G+ and P+G+ collapsed) compared with the no gesture condition (P−G− and P+G− collapsed) in the anterior (*Left*) and posterior (*Right*) ROIs in the N400 (300 to 500 ms) time window (with adjusted baseline correction). Amplitude is reported in microvolts (µV) and time in seconds (s). Negativity is plotted upward. Shaded areas indicate the relevant time windows.

Fig. 2*A* illustrates the grand-average waveforms for each condition across anterior and posterior regions of interest (ROIs) in both time windows (N100: 50 to 150 ms and N400: 300 to 500 ms), with adjusted baseline corrections. Fig. 2*B* shows the topographic distribution across two time windows, with the baseline (P−G−) subtracted from the experimental conditions (P+G+, P−G+, P+G−). Fig. 3*A* illustrates the prosody condition (P+G− and P+G+ collapsed) compared with the no prosody condition (P−G− and P−G+ collapsed) as well as the gesture condition (P−G+ and P+G+ collapsed) compared with the no gesture condition (P−G− and P+G− collapsed).

Visual inspection of the N100 time window revealed that over posterior sites amplitudes were most negative for gesture-present conditions (P−G+ and P+G+) relative to P−G−. This N100 effect indicates an early sensitivity to gestures, likely driven by their inherent perceptual salience (Fig. 2 *A*, *Left*). These gesture-related salience effects are clearly observed when gesture-present conditions are collapsed (irrespective of prosody) and compared with gesture-absent conditions (Fig. 3 *A*, *Right*). No such effect is observed when prosodic conditions are collapsed (irrespective of gesture) and compared with nonprosodic conditions (Fig. 3 *A*, *Left*). To disentangle gesture-related salience effects and potential spillover effects on the N400 time window, a new baseline correction (0 to 200 ms) was applied. Inspection of ERP data with this new baseline indicated an enhanced N400 amplitude in conditions where the gesture was absent, with the largest response observed in P+G− (Fig. 2 *A*, *Right*). Overall, this effect was most pronounced at posterior sites (Fig. 2 *B*, *Right*), and is clearly observed when gesture-present conditions are collapsed (irrespective of prosody) and compared with gesture-absent conditions (Fig. 3 *B*, *Right*).

A linear mixed-effects model was fitted in R (version 4.4.2; R Core Team, 2023) using the lme4 package (lmer function for mixed models) to examine the effects of ROI (anterior and posterior) and Condition (P+G+, P−G+, P+G−, and P−G−) on the mean amplitudes, with Participant as a random intercept. Analyses were conducted on ERP amplitudes averaged across trials, and item-level random effects were therefore not estimated. In the first time window (N100), there were significant main effects of ROI (*P* < 0.001) and Condition (*P* < 0.001) as well as ROI × Condition interaction (*P* < 0.001), primarily driven by the Posterior × P−G+ and Posterior × P+G+ contrasts. This indicates that amplitude modulation by condition was specific to the posterior region, with conditions P−G+ and P+G+ eliciting larger responses. In the second time window (N400), there were significant main effects of ROI (*P* < 0.001) and Condition (*P* < 0.001). However, the ROI × Condition interaction did not reach significance (*P* > 0.05).

Post hoc analyses for the ROI × Condition interaction in the N100 time window indicated that amplitudes showed modest but statistically significant modulation across conditions in the anterior ROI. Compared to the baseline P−G− condition, only P+G+ elicited statistically significant negativity (β = 0.38, t(1505) = 2.70, *P* = 0.035). P+G+ also differed from P+G− (β = 0.61, t(1505) = 4.33, *P* < 0.001) and P−G+ (β = 0.38, t(1505) = 2.72, *P* = 0.033). In the posterior ROI, amplitudes showed a graded difference across conditions where all contrasts were highly significant. Compared to the baseline P−G− condition, amplitudes were more negative in P−G+ (β = 1.09, t(1505) = 7.72, *P* < 0.001) and P+G+ (β = 1.54, t(1505) = 10.95, *P* < 0.001), but less negative in P+G− (β = −0.53, t(1505) = −3.73, *P* = 0.001). Further comparisons confirmed differences between P+G− and P−G+ (β = 1.61, t(1505) = 11.45, *P* < 0.001), between P+G− and P+G+ (β = 2.06, t(1505) = 14.68, *P* < 0.001) and between P−G+ and P+G+ (β = 0.45, t(1505) = 3.23, *P* = 0.007). To recapitulate, in both anterior and posterior ROIs, the largest N100 response occurred for P+G+. Relative to anterior ROI, posterior ROI showed strong condition-dependent changes, with amplitudes shifting from positive in conditions P−G− and P+G− to more negative in conditions P−G+ and P+G+.

Post hoc analyses for the main effect of ROI in the N400 time window indicated that across conditions, the posterior ROI showed larger N400 negativity compared to the anterior ROI (*P* < 0.001), consistent with prior literature on the scalp distribution of the N400. Follow-up analyses for the main effect of Condition in the N400 time window indicated a robust P+G− effect, eliciting significantly larger negativities than both P−G+ (β = −0.35, t(1505) = −2.78, *P* = 0.028) and P+G+ (β = −0.67, t(1505) = −2.52, *P* < 0.001). Additionally, a significant difference was observed between the two extremes of the experimental conditions, P−G− vs. P+G+ (β = −0.41, t(1505) = −3.20, *P* = 0.007), while all other pairwise comparisons were not significant (*P* > 0.05). These results suggest that the presence of gestures influences negation processing, with gesture conditions (G+) reducing N400 negativities relative to conditions without gestures, that is prosody-only conditions.

## Discussion

Negation, as a core aspect of human cognition and grammar, has attracted considerable scholarly attention in the fields of linguistics and logic. The existing neuroscientific studies have largely focused on pragmatic, semantic, and world-knowledge aspects of negation (26, 27). Yet expression of negation at the grammatical level as in standard verbal negation, and its neural underpinnings have not been investigated in multimodal experimental paradigms. Given that language is inherently multimodal (13, 28, 29) and gestures can be considered to be part of grammar (30), this electrophysiological study aimed to examine whether gestures and prosodic marking associated with negation are integral to the processing of negation.

To address this question, EEG data were collected while participants processed standard verbal negation accompanied by gestures and prosodic marking presented alone or in combination. The target language was Turkish. Due to its typologically distinctive grammatical encoding of negation postverbally, together with prosodic and gestural patterns specifically associated with and preceding morphological marking of negation, Turkish provides a valuable case study for examining multimodal nature of negation. This makes it possible not only to explore how the brain processes negation in a typologically distinct language but also to gain broader insights into the mechanisms underlying multimodal grammar.

The earliest neural effect after verb onset was a negative deflection between 50 ms and 150 ms, most likely reflecting modulation of the N100 component. The N100, a negative peak occurring approximately 100 ms after stimulus onset, has been observed across a wide range of sensory tasks, and is thought to reflect, among other functions, stimulus-driven attentional orienting (31–34). In the visual domain, for example, beat gestures have been shown to modulate early ERP effects with a posterior distribution, and are argued to direct attention to the relevant input (35, 36). Notably, Dimitrova et al. (36) reported no N100 differences between beat gestures and grooming movements, linking this finding to general discrimination of visual input. Consistent with these results, the present study observed N100 modulations for stimuli accompanied by gestures. This may reflect the greater perceptual salience of gestures compared to prosodic modulations, likely due to their cross-modal nature and inherently higher attentional prominence in this context.

The second neural deflection occurred between 300 ms and 500 ms is considered to reflect an N400 effect. The N400 is a posterior negativity peaking around 400 ms, elicited by processes of semantic integration. The more difficult a stimulus is to integrate semantically, the larger the N400 amplitude becomes (37, 38). Previous gesture–speech integration studies have shown modulations of the N400 component (e.g., refs. 39–43). For instance, observing iconic gestures (e.g., a drink gesture) facilitates comprehension of upcoming semantically related words (e.g., drink), as reflected by a reduced N400 amplitude on the word (41). Similarly, prosody and meaningful gestures have been found to reduce N400 amplitude, especially for less predictable words (42). Here, we demonstrate a comparable effect in grammatical marking, with a reduction in N400 amplitude in the presence of a negation gesture. It is important to note that speakers tend to produce more gestures during cognitively or linguistically demanding tasks (44). Given that processing negation is generally challenging and requires greater cognitive effort (45; cf. 27), gestures may help mitigate this burden by facilitating the communication of negation, consistent with previous research demonstrating the enhancing role of gestures in speech processing (39–43).

One might question the absence of any facilitatory effect of prosodic prominence without the accompanying gestures, given their established contribution to multiple levels of linguistic processing in general and in Turkish (17, 18, 21) and prior behavioral studies showing how prosody and gestures jointly shape negation interpretation (9). Nonetheless, it is important to note that prosody alone does not signal negation. In Turkish, lexical stress usually falls on the final syllable, irrespective of morphological complexity or syllable weight. However, this pattern is not absolute. In morphologically complex words, certain suffixes and particles attract stress to the immediately preceding syllable. This is the case with the negation suffix -mA, as discussed and examined in the present paper, where stress falls on the preceding stem syllable, as in káz-ma-dı [dig-NEG-PST.3SG] “S/he did not dig.” The same pattern is also observed with the interrogative particle -mI, as in kaz-dí mı [dig-PST.3SG Q] “Did s/he dig?,” where stress falls on the final syllable immediately preceding the question particle. Such a prosodic rule, obligatorily determined by prestressing morphology, may constitute a less reliable cue for negation than a gesture.

Furthermore, it should be kept in mind that in the current study the facilitatory effect of prosody surfaced when it co-occurred with gesture. When both prosody and gesture are present, the processing of negation is facilitated, as indexed by the largest N400 amplitude reduction in comparison to conditions in which both are absent. Notably, gestures, regardless of whether they co-occur with prosodic prominence, consistently facilitated the processing of negation. These findings suggest that gestures play a more independent role in supporting the comprehension of negation, while the facilitatory impact of prosody appears to be contingent upon its integration with gestural information. It is crucial to note that no instances were found of the backward hand flip occurring independently of negation speech contexts (in silence) in our corpus, suggesting it does not function as a standalone negation marker but rather as part of a multimodal negation construction with -mA. Accordingly, it may serve as a reliable anticipatory cue for verbal negation in line with multimodal construction grammar approaches (46).

This pattern of findings indicates that multimodal alignment, specifically, the integration of prosody and gesture enhances the processing of negation in line with the MaOP principle (24). This is the case when gesture and prosody precede negation in verb final languages with postverbal negation marking. To put it differently, processing efficiency is maximized by the early expression of prosodic prominence and a negation gesture in the case of the late arrival of morphological negation marking. Taken together, these findings highlight the distinct yet complementary roles of prosody and gesture in negation processing, as well as their dynamic interaction, with significant typological implications for understanding multimodal communication in Turkish and beyond.

Given that negation gestures differ across languages and cultures (7, 8) (i.e., manual and/or nonmanual), future research should examine languages with different gestural markers as well as with different syntactic and morphological negation strategies with different word orders. Beyond these core markers, less common strategies also exist (1, 19), as in some African languages where prosody (e.g., tonal changes) can signal negation even in the absence of overt markers (20). How gestures interact with tonal negation in such languages remains an open question. Notably, sign languages differ from spoken languages in their linguistic encoding of negation as well as in their prosodic marking. For instance, unlike spoken languages, sign languages often place negative particles (mostly when manual) at the end of the clause rather than following the cross-linguistic tendency of placing them before the verb, and signal negation with prosodic markers such as eye brow movements and head movements preceding the negative particles (47). It is, therefore, warranted to examine different languages to investigate not only how negation is encoded cross-linguistically and cross-modally but also how it is reinforced and interpreted through prosody and gesture when both are in the visual modality, offering broader insights into the neural mechanisms of multimodal language processing.

In conclusion, this study shows that the processing of negation is an orchestration of morphological marking, negation gesture, and to some extent prosodic marking. This multimodal integration might be especially evolved to facilitate processing, given the postverbal positioning of morphological negation marker in Turkish. The present study is an empirical demonstration that gestures and prosodic cues are integrated to facilitate the neural processing of grammatical features, specifically negation. The present data cannot completely rule out anticipatory predictive effects, given that prediction and integration are closely intertwined processes. However, the short temporal interval between the multimodal cues and the negation marker provides stronger support for an integration than a prediction account. The present data offer a significant expansion of prior research on multimodal language processing, which has largely concentrated on the lexical-semantic integration rather than the morphosyntactic level. Further descriptive and EEG research should investigate this multimodal integration across typologically different languages, systematically examine a broader range of manual and nonmanual gestures, in isolation and combined, and test whether similar patterns extend to other forms of negation.

Overall, the findings we report here indicate that language processing is inherently multimodal (i.e., integrating expressions across modalities), and are consistent with proposals that gestures are an integral part of grammar. In these views, it is argued that structures and processes that are typically identified as “grammatical” in verbal expressions (e.g., in construction grammar approaches) may reflect more general organizing principles that also shape the form and function of gestures in multimodal expression (29, 48–49).

## Materials and Methods

### Ethics Statement.

The experiment reported in the present paper was conducted in compliance with the ethical guidelines for human subject research, and the experimental protocol was approved by the Ethics Board of the Social Sciences Faculty of Radboud University (Project code: ECSW-LT-2024-3-6-97160). Participants provided fully informed written consents prior to data collection.

### Participants.

Twenty-four native speakers of Standard Turkish (18 female, 6 male; age range 20 to 31 y, M = 26.3, SD = 4.02) were recruited via flyers posted on public bulletin boards around the Max Planck Institute for Psycholinguistics and Radboud University in Nijmegen and through social media posts among Turkish communities in The Netherlands [April–May, 2024]. The sample size is justified by parity with previous ERP literature relevant to this study (e.g., refs. 35 and 43). All participants were born and raised in Türkiye and reported no history of developmental problems, including vision, hearing, or language. They received €19 as compensation for their participation.

### Stimuli.

**Fig. 4.**
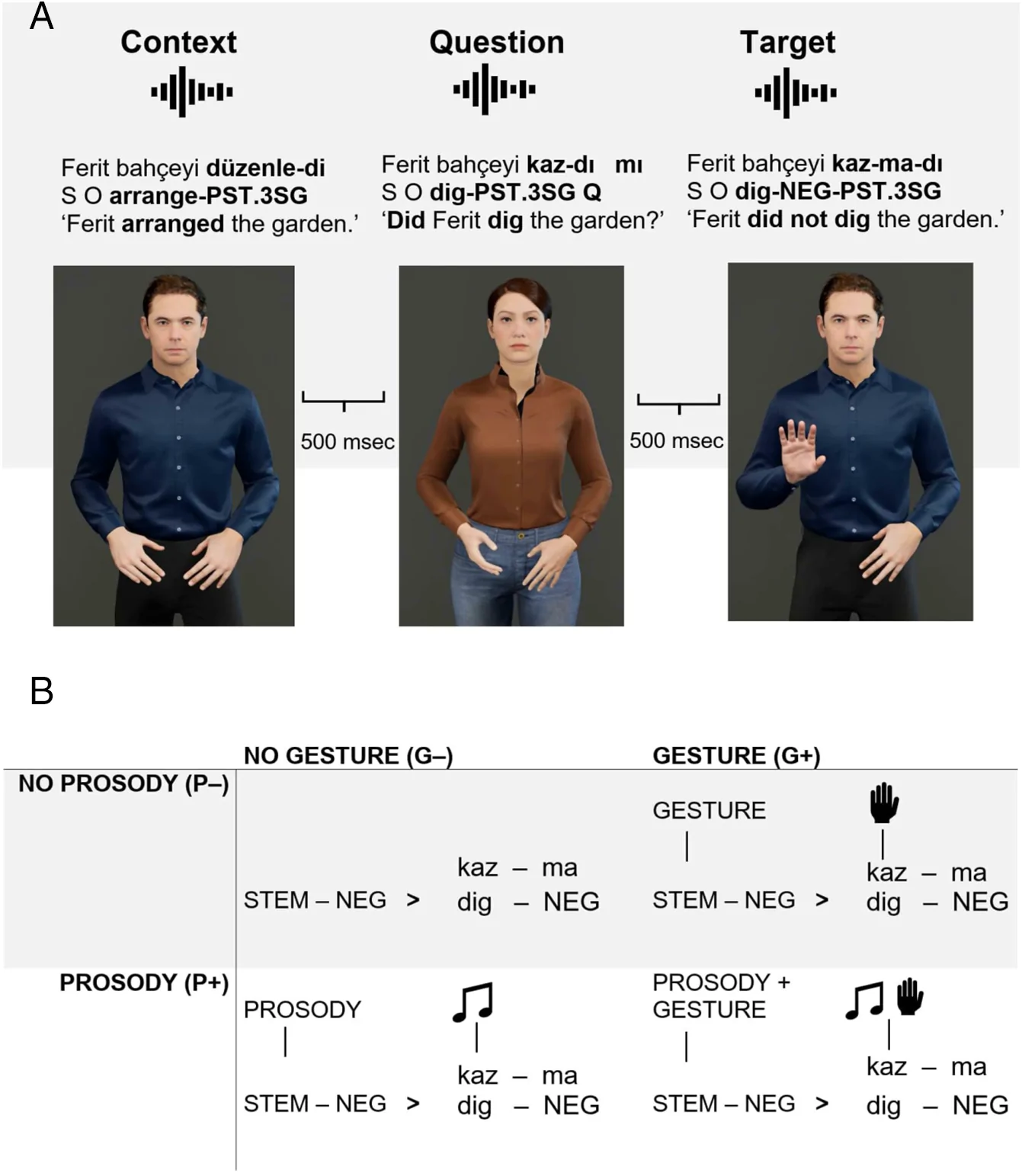
Overview of the experimental stimuli and paradigm. (*A*) One example from an experimental trial embedded in a discourse, consisting of a context sentence, a YES/NO question (with a beat gesture), and a target sentence (with negation gesture). Only verbs are glossed, following the Leipzig Glossing Rules, with English translations provided. Schematic representation of a single trial of the EEG paradigm, with virtual avatars producing each sentence. Time is indicated in milliseconds (ms), and each sentence was separated by a silent period (500 ms). (*B*) Illustration of the experimental conditions, with prosody either present (P+) or absent (P–), and gesture either present (G+) or absent (G–). NEG abbreviation: negation; Note sign: prosody; Hand sign: gesture.

The stimuli consisted of 120 short dialogs in Turkish, each containing three sentences. For a complete set of stimuli, we refer to the Supporting Information. The first sentence introduced a neutral context (not predicting a negation answer), and the second presented a YES/NO question, establishing a narrow focus on a critical word in the third sentence (Fig. 4*A*). The critical words were negated verbs formed using the prestressing negation suffix –mA attached to monosyllabic verb stems, as in káz-ma [dig–NEG] “do not dig.” All critical items appeared in verb-final position, reflecting Turkish’s SOV word order. It is crucial to note that negation in Turkish can also be expressed in verb-only utterances with omitted arguments. The full-sentence forms in target sentences, rather than just verbs with omitted arguments, are included to obtain appropriate neural baselines against which manipulation effects can be assessed. It is also possible that word order may vary, as in prohibition negation (e.g., elle-mé onu [touch–NEG 3SG.ACC] “do not touch it”). However, such word-order variation was not the focus of the present study and was therefore not included in the stimuli items. Investigating this variation would address different research questions and require a different experimental design and neural time-locking approach. Prosody (P) and gesture (G) manipulations were applied directly to the verb stems, with prosody either present (P+) or absent (P–) and gesture either present (G+) or absent (G–), resulting in four experimental conditions: P+G+, P+G–, P–G+, and P–G– (Fig. 4*B*).

Experimental conditions were grouped into four lists according to the Latin square procedure. In each list, an equal number of discourses (30 items) was presented per experimental condition, and no discourses were repeated across the conditions, making 120 sentences per list. To ensure the variability and opaqueness of the experimental material, 90 filler dialogs were included in each list. These filler dialogs followed the same discourse structure, 30 featuring a palm-up gesture with negative sentences, 30 with a palm-up gesture with positive sentences, and 30 with no gesture and positive sentences. For a complete set of filler dialogs, see the Supporting Information.

The specific form of negation gesture was determined based on corpus findings. The corpus consisted of free-spoken dialogs in Turkish extracted from YouTube videos (25). Special attention is given to the backward hand flip, a potentially geographically and culturally specific negation gesture in Turkish. Since our focus is on the morphosyntactic marker, we selected sentences with verbs negated with the suffix -mA, central to the expression of standard verbal negation in Turkish. We excluded sentences containing additional negation elements such as “yok” (indicating absence, e.g., “there is not”), and “hiç” (emphasizing complete absence or denial, e.g., “not at all” or “never”) alongside -mA. We also excluded sentences with copular negation “değil” which negates nominal and adjectival predicates (e.g., it is not green), and is not a morphosyntactic marker.

Consequently, a total of 211 instances of -mA were identified from 87 different speakers. Gestures were annotated using ELAN and categorized into manual only (e.g., backward hand flips), nonmanual only (e.g., backward head tilts), those combining both manual and nonmanual gestures (backward hand flips and backward head tilts), and others (head shakes and lateral hand movements). Gestures appeared in 84.8% of cases (179 out of 211). Among these, 40.2% were accompanied by backward hand flips only (i.e., without any accompanying nonmanual gestures), 35.8% with both manual and nonmanual gestures, 15.6% with nonmanual gestures only, and 8.4% with other negation gestures such as head shakes and lateral hand movements. We took the most frequent form of negation gesture, backward hand flip only accompanying negation sentences with -mA as a model to design our stimuli. An example of such negation gesture from the corpus is provided in *SI Appendix*.

The stimuli were presented using digital avatars created with Unreal Engine’s MetaHuman Creator (version 2023.1; Epic Games, 2023) (Fig. 4*A*). The avatars were designed with neutral physical traits to avoid features that could bias participant responses. The avatars were animated to perform backward hand flips, with gesture strokes timed to begin approximately 400 ms before the verbal negation markers. Gesture strokes overlap with the verb stems, which are always monosyllabic and on average 250 ms long, resulting in gesture strokes starting approximately 150 ms before the verb. The gesture-related modulations are directly aligned with the segment that carries the relevant prosodic information, supporting their comparability in the same linguistic window. The timings were determined based on 35 clear instances from the corpus reported in ref. 50.

Voice recordings were generated with ElevenLabs (2023), with iterations adding prominence (i.e., higher *f*0 and intensity) on syllables preceding negation; additional adjustments were made in Praat (version 6.3.01) when needed to match human-like prosody marking on the verb. These recordings were reviewed by native Turkish speakers to ensure that the intended realizations and manipulations were achieved. The final animations were rendered in Unreal Engine at high resolution to preserve the avatars’ lifelike quality and were verified by the authors. Two types of virtual avatars were created, with context and target sentences delivered by a male avatar and question sentences produced by a female avatar.

### Procedure.

The experiment took place in an electrically shielded and sound-attenuated recording booth. The experimental paradigm was programmed in Presentation (Version 22.1; Psychology Software Tools), and the auditory stimuli were delivered via loudspeakers (Yamaha, HTR-4072). The experiment included a total of 210 trials, comprising 120 from the main experimental conditions and 90 filler trials. Participants were instructed to fixate on a fixation cross displayed for 500 ms after each question, and the inter-trial-interval was set to 1,500 ms. The task of the participants was to listen to the dialogs for comprehension. The experiment, including breaks and electrode application, lasted ∼1.5 h.

### EEG Recording and Analysis.

EEG data were acquired using a BrainAmps DC amplifier system (Brain Products) at a sampling rate of 1,000 Hz. Online recordings were referenced to the left mastoid and band-pass filtered at 0.02 to 100 Hz (8 s time constant). Data were collected from 32 electrodes positioned according to the standard 10 to 20 ActiCAP montage, while horizontal and vertical eye movements were monitored via EOG electrodes.

For offline processing, EEG data were analyzed in BrainVision Analyzer (Version 2.2.0; Brain Products). The continuous signal was first subjected to a zero-phase Butterworth band-pass filter (0.5 to 30 Hz) and a 50 Hz notch filter to reduce line noise. Signals were then rereferenced to the averaged left and right mastoids. Subsequently, the data were segmented into epochs from −500 to 1,000 ms relative to the onset of the critical verbs. The preonset interval (−500 to 0 ms) was used for baseline correction. To detect and reject artifacts, an independent component analysis was performed (Infomax Restricted ICA, 512 steps; rejected eye artifact related components: 1.58 ± 0.58). Any epochs containing activation exceeding ±100 μV were automatically removed (rejected trials P+G+: 0.33 ± 0.86; P−G+: 0.33 ± 0.70; P+G−: 0.54 ± 0.93; P−G−: 0.08 ± 0.28).

ERP waveforms were time-locked to the onset of the sentence-final verbs. Average ERP amplitudes were calculated in two time windows (50 to 150 ms and 300 to 500 ms respectively) that captured the N100 and N400 components. Statistical analyses were done in two ROIs: Anterior (F3, Fz, F4, FC1, FC2, C3, Cz, C4) and Posterior (P3, Pz, P4, CP1, CP2, O1, Oz, O2). The anterior–posterior distinction was motivated by the morphology of the components of interest in this study. In accordance with a hypothesis-driven, rather than data-driven, approach, the time windows and ROIs were defined a priori based on previous literature (e.g., refs. 33, 35, 36, 38, and 42) and subsequently confirmed by visual inspection of the grand averages and topographical maps.

## Supplementary Material

Appendix 01 (PDF)

Movie S1. An example video showing the backward hand flip. Used with permission from info@turkeyvideonetwork.com. The backward hand flip is culture-specific and displays morpheme-like behavior in the grammatical encoding of negation in Turkish.

## Data Availability

Some study data available. Data cannot be shared publicly due to ethical and data protection regulations. Further information and data are available from the Max Planck Institute for Psycholinguistics Data Protection Coordinator (privacy@mpi.nl) for researchers who meet the criteria for access to confidential data.
