# Supplementary material for: Multimodal integration supports neural processing of grammatical negation
Source: Proc Natl Acad Sci U S A. 2026 Sep 16;123(38):e2605004123. doi: 10.1073/pnas.2605004123 (PMC13598220; doi:10.1073/pnas.2605004123)
Supplement: Supplementary file 1 [file pnas.2605004123.sapp.pdf]

PNAS

Supporting Information for
Multimodal integration supports neural processing of grammatical negation

Hatice Zora[1,2]*, Peter Hagoort[1,2], Aslı Özyürek[1,2]

[1] Max Planck Institute for Psycholinguistics, Nijmegen 6525 XD, The Netherlands

[2] Donders Institute for Brain, Cognition and Behaviour, Centre for Cognitive Neuroimaging, Radboud University, Nijmegen 6500 HB, The Netherlands.

* Hatice Zora

Email: Hatice.Zora@mpi.nl

This PDF file includes:

Supporting text (Stimuli)
Legend for Movie S1

Other supporting materials for this manuscript include the following:

Movie S1

Supporting Information Text

Stimuli

Experimental Items with English Translations

For illustrative purposes, the first example has been fully glossed. All remaining negation forms follow the same structural pattern (monosyllabic verb stem–NEG–PAST).

1. Can otelin havuzuna girdi.
   Can entered the hotel's swimming pool.
   Can kolluk taktı mı?
   Did Can put on arm floaties?
   Can kolluk takmadı.
   Can arm floaties put on-NEG-PAST
   Can did not put on arm floaties.
2. Eren köye uğradı.
   Eren visited the village.
   Eren at sürdü mü?
   Did Eren ride a horse?
   Eren at sürmedi.
   Eren did not ride a horse.
3. Yasin davete katıldı.
   Yasin attended the invitation.
   Yasin piyano çaldı mı?
   Did Yasin play the piano?
   Yasin piyano çalmadı.
   Yasin did not play the piano.
4. Elif yemek pişirdi.
   Elif cooked food.
   Elif önlük giydi mi?
   Did Elif wear an apron?
   Elif önlük giymedi.
   Elif did not wear an apron.
5. Ayşe dışarı çıktı.
   Ayşe went outside.
   Ayşe şemsiye aldı mı?
   Did Ayşe take an umbrella?
   Ayşe şemsiye almadı.
   Ayşe did not take an umbrella.
6. Emre kahvaltı yaptı.
   Emre had breakfast.
   Emre yumurta yedi mi?
   Did Emre eat eggs?
   Emre yumurta yemedi.
   Emre did not eat eggs.
7. Deniz futbol oynadı.
   Deniz played football.
   Deniz ayağını burktu mu?
   Did Deniz sprain his ankle?
   Deniz ayağını burkmadı.
   Deniz did not sprain his ankle.
8. Murat börek pişirdi.
   Murat baked börek.
   Murat susam serpti mi?
   Did Murat sprinkle sesame seeds?
   Murat susam serpmedi.
   Murat did not sprinkle sesame seeds.
9. Can misafir ağırladı.

Can hosted guests.
Can şarap açtı mı?
Did Can open wine?
Can şarap açmadı.
Can did not open wine.

10. Hasan hesap ödedi.
    Hasan paid the bill.
    Hasan bahşiş verdi mi?
    Did Hasan leave a tip?
    Hasan bahşiş vermedi.
    Hasan did not leave a tip.
11. Nil evi temizledi.
    Nil cleaned the house.
    Nil çamaşır serdi mi?
    Did Nil hang the laundry?
    Nil çamaşır sermedi.
    Nil did not hang the laundry.
12. Hande kıyafet tasarladı.
    Hande designed clothes.
    Hande gömlek çizdi mi?
    Did Hande draw a shirt?
    Hande gömlek çizmedi.
    Hande did not draw a shirt.
13. Osman tarlada çalıştı.
    Osman worked in the field.
    Osman ekin biçti mi?
    Did Osman harvest the crops?
    Osman ekin biçmedi.
    Osman did not harvest the crops.
14. Aylin Anadolu'yu gezdi.
    Aylin traveled around Anatolia.
    Aylin Çorum'a gitti mi?
    Did Aylin go to Çorum?
    Aylin Çorum'a gitmedi.
    Aylin did not go to Çorum.
15. Onur alışveriş merkezine gitti.
    Onur went to the shopping mall.
    Onur mont baktı mı?
    Did Onur look at jackets?
    Onur mont bakmadı.
    Onur did not look at jackets.
16. Mehmet akşam yemeği yedi.
    Mehmet had dinner.
    Mehmet mum yaktı mı?
    Did Mehmet light a candle?
    Mehmet mum yakmadı.
    Mehmet did not light a candle.
17. Ezgi hayvanat bahçesine gitti.
    Ezgi went to the zoo.
    Ezgi zebra gördü mü?
    Did Ezgi see a zebra?
    Ezgi zebra görmedi.
    Ezgi did not see a zebra.
18. Barış yatağa uzandı.
    Barış lay down on the bed.
    Barış alarm kurdu mu?

Did Barış set an alarm?
Barış alarm kurmadı.
Barış did not set an alarm.

19. Nazlı dışarı çıktı.
Nazlı went outside.
Nazlı araba sürdü mü?
Did Nazlı drive a car?
Nazlı araba sürmedi.
Nazlı did not drive a car.
20. Asansör yukarı kata çıktı.
The elevator went up to the upper floor.
Asansör üçüncü katta durdu mu?
Did the elevator stop on the third floor?
Asansör üçüncü katta durmadı.
The elevator did not stop on the third floor.
21. Faruk parkta köpek gördü.
Faruk saw a dog in the park.
Faruk köpeği sevdi mi?
Did Faruk pet the dog?
Faruk köpeği sevmedi.
Faruk did not pet the dog.
22. Serpil çantasını karıştırdı.
Serpil anahtar buldu mu?
Serpil anahtar bulmadı.
23. Gökhan altınları sakladı.
Gökhan hid the gold.
Gökhan altınları gömdü mü?
Did Gökhan bury the gold?
Gökhan altınları gömmedi.
Gökhan did not bury the gold.
24. Özlem yemek hazırladı.
Özlem prepared food.
Özlem yaprak sardı mı?
Did Özlem make stuffed vine leaves?
Özlem yaprak sarmadı.
Özlem did not make stuffed vine leaves.
25. Tevfik soba yaktı.
Tevfik lit the stove.
Tevfik odun yardı mı?
Did Tevfik chop wood?
Tevfik odun yarmadı.
Tevfik did not chop wood.
26. Ayten tedavi oldu.
Ayten received treatment.
Ayten emara girdi mi?
Did Ayten get an MRI scan?
Ayten emara girmedi.
Ayten did not get an MRI scan.
27. Fulden örgü ördü.
Fulden knitted.
Fulden örgü söktü mü?
Did Fulden unravel the knitting?
Fulden örgü sökmedi.
Fulden did not unravel the knitting.
28. Haluk dişini çektirdi.
Haluk had his tooth extracted.

Haluk ilaç içti mi?
Did Haluk take medicine?
Haluk ilaç içmedi.
Haluk did not take medicine.

29. İklim çalışanları bilgilendirdi.
İklim informed the employees.
İklim mail attı mı?
Did İklim send an email?
İklim mail atmadı.
İklim did not send an email.
30. Faruk asansöre bindi.
Faruk got into the elevator.
Faruk on ikinci kata çıktı mı?
Did Faruk go up to the 12th floor?
Faruk on ikinci kata çıkmadı.
Faruk did not go up to the 12th floor.
31. Ece bilgi yarışmasına katıldı.
Ece participated in the quiz competition.
Ece son soruyu bildi mi?
Did Ece know the final question?
Ece son soruyu bilmedi.
Ece did not know the final question.
32. Ufuk arkadaşını ziyaret etti.
Ufuk visited his friend.
Ufuk arkadaşında kaldı mı?
Did Ufuk stay at his friend's place?
Ufuk arkadaşında kalmadı.
Ufuk did not stay at his friend's place.
33. Egemen ortada koşturup durdu.
Egemen kept running around in the middle.
Egemen yere düştü mü?
Did Egemen fall to the ground?
Egemen yere düşmedi.
Egemen did not fall to the ground.
34. Özcan tekne turu yaptı.
Özcan went on a boat tour.
Özcan denize daldı mı?
Did Özcan dive into the sea?
Özcan denize dalmadı.
Özcan did not dive into the sea.
35. Füsun sınıfa ödev verdi.
Füsun gave homework to the class.
Füsun sınıfı gruplara böldü mü?
Did Füsun divide the class into groups?
Füsun sınıfı gruplara bölmedi.
Füsun did not divide the class into groups.
36. Enes Kapadokya'ya gitti.
Enes went to Cappadocia.
Enes balona bindi mi?
Did Enes get on a hot air balloon?
Enes balona binmedi.
Enes did not get on a hot air balloon.
37. Halil anten taktı.
Halil installed the antenna.
Halil anteni eğdi mi?
Did Halil bend the antenna?

Halil anteni eğmedi.
Halil did not bend the antenna.

38. Sevcan okula gitti.
Sevcan went to school.
Sevcan saçlarını ördü mü?
Did Sevcan braid her hair?
Sevcan saçlarını örmedi.
Sevcan did not braid her hair.
39. Fatma tatlı yaptı.
Fatma made dessert.
Fatma lokma döktü mü?
Did Fatma make lokma (fried dough dessert)?
Fatma lokma dökmedi.
Fatma did not make lokma.
40. Hanife kuruyemiş koydu.
Hanife put nuts.
Hanife ceviz kırdı mı?
Did Hanife crack walnuts?
Hanife ceviz kırmadı.
Hanife did not crack walnuts.
41. Mahir partnerini dansa kaldırdı.
Mahir invited his partner to dance.
Mahir partnerini öptü mü?
Did Mahir kiss his partner?
Mahir partnerini öpmedi.
Mahir did not kiss his partner.
42. Kenan sokakta yürüdü.
Kenan walked on the street.
Kenan adres sordu mu?
Did Kenan ask for directions?
Kenan adres sormadı.
Kenan did not ask for directions.
43. Yasemin bir çuval soğan aldı.
Yasemin bought a sack of onions.
Yasemin çuvalı tarttı mı?
Did Yasemin weigh the sack?
Yasemin çuvalı tartmadı.
Yasemin did not weigh the sack.
44. Sait Almanya'dan davet aldı.
Sait received an invitation from Germany.
Sait Almanya'ya göçtü mü?
Did Sait migrate to Germany?
Sait Almanya'ya göçmedi.
Sait did not migrate to Germany.
45. Hülya gişeden geçti.
Hülya passed through the ticket booth.
Hülya kart bastı mı?
Did Hülya swipe her card?
Hülya kart basmadı.
Hülya did not swipe her card.
46. Nedim yeni ev aldı.
Nedim bought a new house.
Nedim kredi çekti mi?
Did Nedim take out a loan?
Nedim kredi çekmedi.
Nedim did not take out a loan.

47. Belediye köyü ziyaret etti.
    The municipality visited the village.
    Belediye ağaç dikti mi?
    Did the municipality plant trees?
    Belediye ağaç dikmedi.
    The municipality did not plant trees.
48. Necmiye evi temizledi.
    Necmiye cleaned the house.
    Necmiye cam sildi mi?
    Did Necmiye clean the windows?
    Necmiye cam silmedi.
    Necmiye did not clean the windows.
49. Hakan sevgilisine hediye verdi.
    Hakan gave a gift to his girlfriend.
    Hakan mektup yazdı mı?
    Did Hakan write a letter?
    Hakan mektup yazmadı.
    Hakan did not write a letter.
50. Kedi kule ile oynadı.
    The cat played with the tower.
    Kedi kuleyi yıktı mı?
    Did the cat knock the tower down?
    Kedi kuleyi yıkmadı.
    The cat did not knock the tower down.
51. Melis alışveriş merkezinde dolaştı.
    Melis wandered around the shopping mall.
    Melis küpe seçti mi?
    Did Melis choose earrings?
    Melis küpe seçmedi.
    Melis did not choose earrings.
52. Mimar evi düzenledi.
    The architect arranged the house.
    Mimar resim astı mı?
    Did the architect hang a picture?
    Mimar resim asmadı.
    The architect did not hang a picture.
53. Çocuk odasına girdi.
    The child entered the room.
    Çocuk yatağına yattı mı?
    Did the child lie down on his bed?
    Çocuk yatağına yatmadı.
    The child did not lie down on his bed.
54. Ozan Haliç'te zaman geçirdi.
    Ozan spent time at the Golden Horn.
    Ozan balık tuttu mu?
    Did Ozan go fishing?
    Ozan balık tutmadı.
    Ozan did not go fishing.
55. Serpil temizlik yaptı.
    Serpil did cleaning.
    Serpil halı silkti mi?
    Did Serpil shake the carpet?
    Serpil halı silkmedi.
    Serpil did not shake the carpet.
56. Melike uzun yıllar program yaptı.
    Melike worked in broadcasting for many years.

Melike müzik programı sundu mu?
Did Melike present a music program?
Melike müzik programı sunmadı.
Melike did not present a music program.

57. Süleyman depo düzenledi.
Süleyman organized the warehouse.
Süleyman ürün saydı mı?
Did Süleyman count the products?
Süleyman ürün saymadı.
Süleyman did not count the products.
58. Vildan dolaba meyve yerleştirdi.
Vildan placed fruit in the fridge.
Vildan portakal koydu mu?
Did Vildan put oranges?
Vildan portakal koymadı.
Vildan did not put oranges.
59. Şirket yeni bina dikti.
The company built a new building.
Şirket ağaç kesti mi?
Did the company cut down trees?
Şirket ağaç kesmedi.
The company did not cut down trees.
60. Tren gara yanaştı.
The train arrived at the station.
Tren zamanında kalktı mı?
Did the train depart on time?
Tren zamanında kalkmadı.
The train did not depart on time.
61. Nefise uçaktan korktu.
Nefise was afraid of the plane.
Nefise uçaktan indi mi?
Did Nefise get off the plane?
Nefise uçaktan inmedi.
Nefise did not get off the plane.
62. Kıvılcım çeşit çeşit boncuk aldı.
Kıvılcım bought various beads.
Kıvılcım boncukları ipe dizdi mi?
Did Kıvılcım string the beads onto a thread?
Kıvılcım boncukları ipe dizmedi.
Kıvılcım did not string the beads onto a thread.
63. Başak dışarıda duman gördü.
Başak saw smoke outside.
Başak patlama sesi duydu mu?
Did Başak hear an explosion?
Başak patlama sesi duymadı.
Başak did not hear an explosion.
64. Hilal mutfağı temizledi.
Hilal cleaned the kitchen.
Hilal tencere ovdu mu?
Did Hilal scrub the pot?
Hilal tencere ovmadı.
Hilal did not scrub the pot.
65. İkbal Ege'de tatil yaptı.
İkbal had a holiday in the Aegean region.
İkbal Alaçatı'yı gezdi mi?
Did İkbal visit Alaçatı?

İkbal Alaçatı'yı gezmedi.
İkbal did not visit Alaçatı.

66. Rıza dikiş makinasını açtı.
Rıza turned on the sewing machine.
Rıza kumaş ölçtü mü?
Did Rıza measure the fabric?
Rıza kumaş ölçmedi.
Rıza did not measure the fabric.
67. Ali kümese girdi.
Ali entered the chicken coop.
Ali tavukları saldı mı?
Did Ali let the chickens out?
Ali tavukları salmadı.
Ali did not let the chickens out.
68. Gülçin salata hazırladı.
Gülçin prepared a salad.
Gülçin domates soydu mu?
Did Gülçin peel tomatoes?
Gülçin domastes soymadı.
Gülçin did not peel tomatoes.
69. Nihal bahçede fare gördü.
Nihal saw a mouse in the garden.
Nihal fareden korktu mu?
Did Nihal get scared of the mouse?
Nihal fareden korkmadı.
Nihal did not get scared of the mouse.
70. İrem duvara aksesuar koydu.
İrem put a decoration on the wall.
İrem duvarı deldi mi?
Did İrem drill the wall?
İrem duvarı delmedi.
İrem did not drill the wall.
71. Nazlı içecek hazırladı.
Nazlı prepared a drink.
Nazlı limon sıktı mı?
Did Nazlı squeeze lemons?
Nazlı limon sıkmadı.
Nazlı did not squeeze lemons.
72. Ferit evin çevresini düzenledi.
Ferit organized the area around the house.
Ferit bahçe kazdı mı?
Did Ferit dig the garden?
Ferit bahçe kazmadı.
Ferit did not dig the garden.
73. Baran ders çalıştı.
Baran studied.
Baran geometri sorusu çözdü mü?
Did Baran solve a geometry problem?
Baran geometri sorusu çözmedi.
Baran did not solve a geometry problem.
74. Neslihan kocasını anlattı.
Neslihan talked about her husband.
Neslihan kocasını övdü mü?
Did Neslihan praise her husband?
Neslihan kocasını övmedi.
Neslihan did not praise her husband.

75. Ege köpek ile karşılaştı.
Ege encountered a dog.
Ege köpekten ürktü mü?
Did Ege get scared of the dog?
Ege köpekten ürkmedi.
Ege did not get scared of the dog.
76. Kemal notere gitti.
Kemal went to the notary.
Kemal araba sattı mı?
Did Kemal sell a car?
Kemal araba satmadı.
Kemal did not sell a car.
77. Hülya polisleri gördü.
Hülya saw the police.
Hülya polisten kaçtı mı?
Did Hülya run away from the police?
Hülya polisten kaçmadı.
Hülya did not run away from the police.
78. Melih ağrıdan yakındı.
Melih complained about pain.
Melih ilaç yuttu mu?
Did Melih take medicine?
Melih ilaç yutmadı.
Melih did not take medicine.
79. Pınar sınava girdi.
Pınar took the exam.
Pınar sınavı geçti mi?
Did Pınar pass the exam?
Pınar sınavı geçmedi.
Pınar did not pass the exam.
80. Halil arkadaşları ile buluştu.
Halil met with his friends.
Halil neşe saçtı mı?
Did Halil spread joy?
Halil neşe saçmadı.
Halil did not spread joy.
81. Çocuklar kar topu oynadı.
Children played snowball fights.
Çocuklar buzda kaydı mı?
Did the children slide on the ice?
Çocuklar buzda kaymadı.
The children did not slide on the ice.
82. Hüseyin koltukları yıkadı.
Hüseyin washed the sofas.
Hüseyin örtü yaydı mı?
Did Hüseyin spread the cover?
Hüseyin örtü yaymadı.
Hüseyin did not spread the cover.
83. Sibel bahçeye indi.
Sibel went down to the garden.
Sibel ot yoldu mu?
Did Sibel pull weeds?
Sibel ot yolmadı.
Sibel did not pull weeds.
84. Nazlı yurtdışına gitti.
Nazlı went abroad.

Nazlı Belçika'ya uçtu mu?
Did Nazlı fly to Belgium?
Nazlı Belçika'ya uçmadı.
Nazlı did not fly to Belgium.

85. Demet göle geldi.
Demet came to the lake.
Demet gölde yüzdü mü?
Did Demet swim in the lake?
Demet gölde yüzmedi.
Demet did not swim in the lake.
86. Ergin sos hazırladı.
Ergin prepared sauce.
Ergin sarımsak ezdi mi?
Did Ergin crush garlic?
Ergin sarımsak ezmedi.
Ergin did not crush garlic.
87. Kaan evde koşturdu.
Kaan ran around at home.
Kaan masaya çarptı mı?
Did Kaan hit the table?
Kaan masaya çarpmadı.
Kaan did not hit the table.
88. Samet kumandayla oynadı.
Samet played with the remote control.
Samet kumandayı bozdu mu?
Did Samet break the remote control?
Samet kumandayı bozmadı.
Samet did not break the remote control.
89. Burak teli taktı.
Burak installed the wire.
Burak teli büktü mü?
Did Burak bend the wire?
Burak teli bükmedi.
Burak did not bend the wire.
90. Buse yazı Akdeniz'de geçirdi.
Buse spent the summer in the Mediterranean.
Buse sıcaktan bezdi mi?
Did Buse get tired of the heat?
Buse sıcaktan bezmedi.
Buse did not get tired of the heat.
91. Sinek odada iki tur dolaştı.
The fly wandered around the room for two rounds.
Sinek masaya kondu mu?
Did the fly land on the table?
Sinek masaya konmadı.
The fly did not land on the table.
92. Pınar başkanı dinledi.
Pınar listened to the president.
Pınar başkanın niyetini sezdi mi?
Did Pınar sense the president's intention?
Pınar başkanın niyetini sezmedi.
Pınar did not sense the president's intention.
93. Papağan eve geldi.
The parrot came home.
Papağan gece öttü mü?
Did the parrot chirp at night?

Papağan gece ötmedi.
The parrot did not chirp at night.

94. Ufuk pişti oynadı.
Ufuk played poker.
Ufuk kartları kardı mı?
Did Ufuk shuffle the cards?
Ufuk kartları karmadı.
Ufuk did not shuffle the cards.
95. Özcan duvarı boyadı.
Özcan painted the wall.
Özcan çivi çaktı mı?
Did Özcan hammer a nail?
Özcan çivi çakmadı.
Özcan did not hammer a nail.
96. Sezen dizi izledi.
Sezen watched a TV series.
Sezen diziden bıktı mı?
Did Sezen get bored of the series?
Sezen diziden bıkmadı.
Sezen did not get bored of the series.
97. Şirket krizden etkilendi.
The company was affected by the crisis.
Şirket krizde battı mı?
Did the company go bankrupt in the crisis?
Şirket krizde batmadı.
The company did not go bankrupt in the crisis.
98. Güliz yumurta pişirdi.
Güliz cooked eggs.
Güliz yumurta çırptı mı?
Did Güliz whisk the eggs?
Güliz yumurta çırpmadı.
Güliz did not whisk the eggs.
99. Cem ipi astı.
Cem hung the rope.
Cem ipi gerdi mi?
Did Cem tighten the rope?
Cem ipi germedi.
Cem did not tighten the rope.
100. Selin sütü kaynattı.
Selin boiled the milk.
Selin sütü süzdü mü?
Did Selin strain the milk?
Selin sütü süzmedi.
Selin did not strain the milk.
101. Zeytinyağı şişede bekledi.
Olive oil stayed in the bottle.
Zeytinyağı şişeden sızdı mı?
Did the olive oil leak from the bottle?
Zeytinyağı şişeden sızmadı.
The olive oil did not leak from the bottle.
102. Kumaş iki kez yıkandı.
The fabric was washed twice.
Kumaş çok soldu mu?
Did the fabric fade a lot?
Kumaş çok solmadı.

The fabric did not fade a lot.

103. Yılmaz yemek yedi.
Yılmaz ate food.
Yılmaz yediğini kustu mu?
Did Yılmaz vomit what he ate?
Yılmaz yediğini kusmadı.
Yılmaz did not vomit what he ate.

104. Havuz su aldı.
The pool took in water.
Havuz tam doldu mu?
Did the pool fill completely?
Havuz tam dolmadı.
The pool did not fill completely.

105. Tarık patlıcan pişirdi.
Tarık cooked eggplant.
Tarık patlıcanın içini oydu mu?
Did Tarık hollow out the eggplant?
Tarık patlıcanın içini oymadı.
Tarık did not hollow out the eggplant.

106. Reyhan sakız çiğnedi.
Reyhan chewed gum.
Reyhan sakızı yuttu mu?
Did Reyhan swallow the gum?
Reyhan sakızı yutmadı.
Reyhan did not swallow the gum.

107. Veli taş topladı.
Veli collected stones.
Veli taşları yonttu mu?
Did Veli carve the stones?
Veli taşları yontmadı.
Veli did not carve the stones.

108. Leyla bağ evine uğradı.
Leyla visited the country house.
Leyla beyaz şarap tattı mı?
Did Leyla taste white wine?
Leyla beyaz şarap tatmadı.
Leyla did not taste white wine.

109. Arif kışa hazırlandı.
Arif prepared for winter.
Arif odun yığdı mı?
Did Arif pile up wood?
Arif odun yığmadı.
Arif did not pile up wood.

110. Irmak toplantıda endişelendi.
Irmak felt anxious in the meeting.
Irmak vücudunu kastı mı?
Did Irmak tense her body?
Irmak vücudunu kasmadı.
Irmak did not tense her body.

111. Halil kırpma makası aldı.
Halil bought pruning shears.
Halil koyun kırptı mı?
Did Halil shear sheep?
Halil koyun kırpmadı.
Halil did not shear sheep.

112. Çağla ani fren yaptı.

Çağla made an emergency brake.
Çağla arabayı sürttü mü?
Did Çağla scratch the car?
Çağla arabayı sürtmedi.
Çağla did not scratch the car.

113. Selim arkadaşını uyardı.
Selim warned his friend.
Selim arkadaşını dürttü mü?
Did Selim poke his friend?
Selim arkadaşını dürtmedi.
Selim did not poke his friend.

114. Bitkiler soğukta kaldı.
Plants stayed in the cold.
Bitkiler soğukta dondu mu?
Did the plants freeze in the cold?
Bitkiler soğukta donmadı.
The plants did not freeze in the cold.

115. Bina depremden etkilendi.
The building was affected by the earthquake.
Bina depremde çöktü mü?
Did the building collapse in the earthquake?
Bina depremde çökmedi.
The building did not collapse in the earthquake.

116. Mahir yolda kaldı.
Mahir was left on the road.
Mahir aracını itti mi?
Did Mahir push his vehicle?
Mahir aracını itmedi.
Mahir did not push his vehicle.

117. Şule ava çıktı.
Şule went hunting.
Şule tavşan vurdu mu?
Did Şule shoot a rabbit?
Şule tavşan vurmadı.
Şule did not shoot a rabbit.

118. Usta duvarı değiştirdi.
The master changed the wall.
Usta duvarı deldi mi?
Did the master drill the wall?
Usta duvarı delmedi.
The master did not drill the wall.

119. Dere yatağı yağıştan etkilendi.
The riverbed was affected by rainfall.
Dere yatağı taştı mı?
Did the riverbed overflow?
Dere yatağı taşmadı.
The riverbed did not overflow.

120. İp ortadan gerildi.
The rope was stretched in the middle.
İp ortadan koptu mu?
Did the rope break in the middle?
İp ortadan kopmadı.
The rope did not break in the middle.

Filler Items

The filler dialogues followed the same discourse structure as the experimental stimuli, 30 featuring a palm-up gesture with negative sentences, 30 with a palm-up gesture with positive sentences, and 30 with no gesture and positive sentences.

30 negative answers with palm up gesture

1. Hakan takım elbise giydi.
   Hakan wore a suit.
   Hakan saat taktı mı?
   Did Hakan wear a watch?
   Hakan saat takmadı.
   Hakan did not wear a watch.
2. Yusuf tarlaya gitti.
   Yusuf went to the field.
   Yusuf tarlayı sürdü mü?
   Did Yusuf plow the field?
   Yusuf tarlayı sürmedi.
   Yusuf did not plow the field.
3. Hülya otobüsü kaçırdı.
   Hülya missed the bus.
   Hülya ıslık çaldı mı?
   Did Hülya whistle?
   Hülya ıslık çalmadı.
   Hülya did not whistle.
4. Sezen davete katıldı.
   Sezen attended the event.
   Sezen etek giydi mi?
   Did Sezen wear a skirt?
   Sezen etek giymedi.
   Sezen did not wear a skirt.
5. Yaren derse girdi.
   Yaren entered the class.
   Yaren beslenme çantası aldı mı?
   Did Yaren bring a lunchbox?
   Yaren beslenme çantası almadı.
   Yaren did not bring a lunchbox.
6. Barış yemek siparişi verdi.
   Barış placed a food order.
   Barış kebap yedi mi?
   Did Barış eat kebab?
   Barış kebap yemedi.
   Barış did not eat kebab.
7. Hande valizleri taşıdı.
   Hande carried the suitcases.
   Hande elini burktu mu?
   Did Hande sprain her wrist?
   Hande elini burkmadı.
   Hande did not sprain her wrist.
8. Yüsra eti fırına verdi.
   Yüsra put the meat in the oven.
   Yüsra kekik serpti mi?
   Did Yüsra sprinkle thyme?
   Yüsra kekik serpmedi.
   Yüsra did not sprinkle thyme.
9. Irmak araba sürdü.
   Irmak drove a car.

Irmak klima açtı mı?
Did Irmak turn on the air conditioner?
Irmak klima açmadı.
Irmak did not turn on the air conditioner.

10. Ufuk arkadaşı ile görüştü.
    Ufuk met with his friend.
    Ufuk borç para verdi mi?
    Did Ufuk lend money?
    Ufuk borç para vermedi.
    Ufuk did not lend money.
11. Pınar balkonu toparladı.
    Pınar tidied the balcony.
    Pınar kilim serdi mi?
    Did Pınar lay a rug?
    Pınar kilim sermedi.
    Pınar did not lay a rug.
12. Hazan yağlı boya tabloyu bitirdi.
    Hazan finished the oil painting.
    Hazan balık çizdi mi?
    Did Hazan draw a fish?
    Hazan balık çizmedi.
    Hazan did not draw a fish.
13. Mahir traktör sürdü.
    Mahir drove a tractor.
    Mahir arpa biçti mi?
    Did Mahir harvest barley?
    Mahir arpa biçmedi.
    Mahir did not harvest barley.
14. Ahmet komşusu ile kavga etti.
    Ahmet fought with his neighbor.
    Ahmet mahkemeye gitti mi?
    Did Ahmet go to court?
    Ahmet mahkemeye gitmedi.
    Ahmet did not go to court.
15. Nevin Afrika tatili yaptı.
    Nevin went on a trip to Africa.
    Nevin Libya'ya gitti mi?
    Did Nevin go to Libya?
    Nevin Libya'ya gitmedi.
    Nevin did not go to Libya.
16. Ali gömlek ütüledi.
    Ali ironed the shirt.
    Ali gömleği yaktı mı?
    Did Ali burn the shirt?
    Ali gömleği yakmadı.
    Ali did not burn the shirt.
17. Gizem dalış yaptı.
    Gizem went diving.
    Gizem vatoz balığı gördü mü?
    Did Gizem see a stingray?
    Gizem vatoz balığı görmedi.
    Gizem did not see a stingray.
18. Semra kışa hazırlık yaptı.
    Semra prepared for winter.
    Semra turşu kurdu mu?
    Did Semra make pickles?

Semra turşu kurmadı.
Semra did not make pickles.

19. Faruk köy evinde kaldı.
    Faruk stayed in the village house.
    Faruk at sürdü mü?
    Did Faruk ride a horse?
    Faruk at sürmedi.
    Faruk did not ride a horse.
20. Otobüs yola çıktı.
    The bus set off.
    Otobüs Ataköy'de durdu mu?
    Did the bus stop at Ataköy?
    Otobüs Ataköy'de durmadı.
    The bus did not stop at Ataköy.
21. Gamze elbise denedi.
    Gamze tried on a dress.
    Gamze elbiseyi sevdi mi?
    Did Gamze like the dress?
    Gamze elbiseyi sevmedi.
    Gamze did not like the dress.
22. Onur birçok sorunla karşılaştı.
    Onur encountered many problems.
    Onur çözüm buldu mu?
    Did Onur find a solution?
    Onur çözüm bulmadı.
    Onur did not find a solution.
23. Kaptan yola çıktı.
    The captain set off.
    Kaptan limana vardı mı?
    Did the captain arrive at the port?
    Kaptan limana varmadı.
    The captain did not arrive at the port.
24. Fatma dikiş dikti.
    Fatma sewed.
    Fatma kırmızı ip sardı mı?
    Did Fatma wind red thread?
    Fatma kırmızı ip sarmadı.
    Fatma did not wind red thread.
25. Kemal bahçe işlerini bitirdi.
    Kemal finished the garden work.
    Kemal odun yardı mı?
    Did Kemal split wood?
    Kemal odun yarmadı.
    Kemal did not split wood.
26. Doktor acilde çalıştı.
    The doctor worked in the emergency room.
    Doktor ameliyata girdi mi?
    Did the doctor enter surgery?
    Doktor ameliyata girmedi.
    The doctor did not enter surgery.
27. Erdem makinayı inceledi.
    Erdem examined the machine.
    Erdem makinayı söktü mü?
    Did Erdem disassemble the machine?
    Erdem makinayı sökmedi.
    Erdem did not disassemble the machine.

28. Kenan karnını doyurdu.
Kenan had a meal.
Kenan şalgam içti mi?
Did Kenan drink turnip juice?
Kenan şalgam içmedi.
Kenan did not drink turnip juice.
29. Leyla tekne ile denize açıldı.
Leyla set sail by boat.
Leyla zincir attı mı?
Did Leyla drop anchor?
Leyla zincir atmadı.
Leyla did not drop anchor.
30. Refik uzun bir yürüyüş yaptı.
Refik went on a long walk.
Refik yokuş çıktı mı?
Did Refik climb the hill?
Refik yokuş çıkmadı.
Refik did not climb the hill.

30 positive answers with palm up gesture

1. Kaan testi bitirdi.
Kaan finished the test.
Kaan tüm cevapları bildi mi?
Did Kaan know all the answers?
Kaan tüm cevapları bildi.
Kaan knew all the answers.
2. Hazan ormana gitti.
Hazan went to the forest.
Hazan ağaç evde kaldı mı?
Did Hazan stay in the tree house?
Hazan ağaç evde kaldı.
Hazan stayed in the tree house.
3. Fikret ağaca çıktı.
Fikret climbed the tree.
Fikret ağaçtan düştü mü?
Did Fikret fall from the tree?
Fikret ağaçtan düştü.
Fikret fell from the tree.
4. Nazan denize girdi.
Nazan entered the sea.
Nazan derine daldı mı?
Did Nazan dive deep?
Nazan derine daldı.
Nazan dove deep.
5. Burcu patatesleri tencereye koydu.
Burcu put the potatoes in the pot.
Burcu patatesleri böldü mü?
Did Burcu cut the potatoes?
Burcu patatesleri böldü.
Burcu cut the potatoes.
6. Gülçin Taksim'de dolaştı.
Gülçin walked around Taksim.
Gülçin tramvaya bindi mi?
Did Gülçin take the tram?
Gülçin tramvaya bindi.

Gülçin took the tram.

7. Mustafa iletken tel aldı.
   Mustafa got a conductive wire.
   Mustafa teli eğdi mi?
   Did Mustafa bend the wire?
   Mustafa teli eğdi.
   Mustafa bent the wire.
8. Usta ön bahçede çalıştı.
   The master worked in the front garden.
   Usta duvar ördü mü?
   Did the master build a wall?
   Usta duvar ördü.
   The master built a wall.
9. Halil kümesi temizledi.
   Halil cleaned the coop.
   Halil yem döktü mü?
   Did Halil pour feed?
   Halil yem döktü.
   Halil poured feed.
10. Nurcan bulaşıkları yerleştirdi.
    Nurcan put the dishes away.
    Nurcan bardak kırdı mı?
    Did Nurcan break a glass?
    Nurcan bardak kırdı.
    Nurcan broke a glass.
11. Ufuk arkadaşını tebrik etti.
    Ufuk congratulated his friend.
    Ufuk arkadaşını öptü mü?
    Did Ufuk kiss his friend?
    Ufuk arkadaşını öptü.
    Ufuk kissed his friend.
12. Eren doktora göründü.
    Eren went to see the doctor.
    Eren rapor sordu mu?
    Did Eren ask for a report?
    Eren rapor sordu.
    Eren asked for a report.
13. Büşra irmik tatlısı yaptı.
    Büşra made semolina dessert.
    Büşra irmiği tarttı mı?
    Did Büşra weigh the semolina?
    Büşra irmiği tarttı.
    Büşra weighed the semolina.
14. Bahar Fransa'yı çok sevdi.
    Bahar loved France very much.
    Bahar Fransa'ya göçtü mü?
    Did Bahar emigrate to France?
    Bahar Fransa'ya göçtü.
    Bahar emigrated to France.
15. Hüseyin asansöre bindi.
    Hüseyin got into the elevator.
    Hüseyin yardım tuşuna bastı mı?
    Did Hüseyin press the help button?
    Hüseyin yardım tuşuna bastı.
    Hüseyin pressed the help button.
16. Engin bankacı ile görüştü.

Engin met with the banker.
Engin para çekti mi?
Did Engin withdraw money?
Engin para çekti.
Engin withdrew money.

17. Selin dikiş makinasını açtı.
Selin opened the sewing machine.
Selin süveter dikti mi?
Did Selin knit a sweater?
Selin süveter dikti.
Selin knitted a sweater.
18. Öğrenci sözlüye kalktı.
The student went up for the oral exam.
Öğrenci tahtayı sildi mi?
Did the student erase the board?
Öğrenci tahtayı sildi.
The student erased the board.
19. Taner Türkçe ödevlerini yaptı.
Taner did his Turkish homework.
Taner şiir yazdı mı?
Did Taner write a poem?
Taner şiir yazdı.
Taner wrote a poem.
20. Deprem sabah saatlerinde oldu.
The earthquake happened in the morning.
Deprem binaları yıktı mı?
Did the earthquake destroy buildings?
Deprem binaları yıktı.
The earthquake destroyed buildings.
21. Eda teknoloji mağazasına gitti.
Eda went to the electronics store.
Eda kulaklık seçti mi?
Did Eda choose headphones?
Eda kulaklık seçti.
Eda chose headphones.
22. Buket fotoğrafları bastırdı.
Buket printed the photos.
Buket fotoğrafları astı mı?
Did Buket hang the photos?
Buket fotoğrafları astı.
Buket hung the photos.
23. Zeliha dedesinin evine geldi.
Zeliha came to her grandfather's house.
Zeliha divana yattı mı?
Did Zeliha lie on the sofa?
Zeliha divana yattı.
Zeliha lay on the sofa.
24. Orhun söz verdi.
Orhun gave his word.
Orhun sözünü tuttu mu?
Did Orhun keep his promise?
Orhun sözünü tuttu.
Orhun kept his promise.
25. Serkan kilimleri topladı.
Serkan collected the rugs.
Serkan kilimleri silkti mi?

Did Serkan shake the rugs?
Serkan kilimleri silkti.
Serkan shook the rugs.

26. Ece çalışmasını bitirdi.
    Ece finished her work.
    Ece çalışmasını sundu mu?
    Did Ece present her work?
    Ece çalışmasını sundu.
    Ece presented her work.
27. Sait yürüyüşe çıktı.
    Sait went for a walk.
    Sait yürüyüş süresini saydı mı?
    Did Sait time the walk?
    Sait yürüyüş süresini saydı.
    Sait timed the walk.
28. Elif kahve hazırladı.
    Elif prepared coffee.
    Elif kahveye süt koydu mu?
    Did Elif put milk in the coffee?
    Elif kahveye süt koydu.
    Elif put milk in the coffee.
29. Polis kontrole çıktı.
    The police went on patrol.
    Polis ceza kesti mi?
    Did the police issue a fine?
    Polis ceza kesti.
    The police issued a fine.
30. Kayhan gösteriyi alkışladı.
    Kayhan applauded the show.
    Kayhan ayağa kalktı mı?
    Did Kayhan stand up?
    Kayhan ayağa kalktı.
    Kayhan stood up.

30 positive answers without gesture

1. Satıcı perakende satış yaptı.
   The seller made a retail sale.
   Satıcı fiyatı indi mi?
   Did the price go down?
   Satıcı fiyatı indi.
   The price went down.
2. Berrin kadehleri yıkadı.
   Berrin washed the glasses.
   Berrin kadehleri dizdi mi?
   Did Berrin arrange the glasses?
   Berrin kadehleri dizdi.
   Berrin arranged the glasses.
3. Gül dışarıyı izledi.
   Gül watched outside.
   Gül ses duydu mu?
   Did Gül hear a sound?
   Gül ses duydu.
   Gül heard a sound.
4. Mehmet bulaşıkları yıkadı.
   Mehmet washed the dishes.

Mehmet çaydanlığı ovdu mu?
Did Mehmet scrub the teapot?
Mehmet çaydanlığı ovdu.
Mehmet scrubbed the teapot.

5. Seçkin tesise girdi.
   Seçkin entered the facility.
   Seçkin imalathaneyi gezdi mi?
   Did Seçkin tour the factory?
   Seçkin imalathaneyi gezdi.
   Seçkin toured the factory.
6. Bahriye rengarenk kumaşlar aldı.
   Bahriye bought colorful fabrics.
   Bahriye kumaşları ölçtü mü?
   Did Bahriye measure the fabrics?
   Bahriye kumaşları ölçtü.
   Bahriye measured the fabrics.
7. Nuri ahıra girdi.
   Nuri entered the barn.
   Nuri inekleri saldı mı?
   Did Nuri release the cows?
   Nuri inekleri saldı.
   Nuri released the cows.
8. Berk meyve tabağı hazırladı.
   Berk prepared a fruit plate.
   Berk elma soydu mu?
   Did Berk peel the apple?
   Berk elma soydu.
   Berk peeled the apple.
9. Nermin karanlıkta yürüdü.
   Nermin walked in the dark.
   Nermin karanlıktan korktu mu?
   Did Nermin get scared of the dark?
   Nermin karanlıktan korktu.
   Nermin was afraid of the dark.
10. Mümin dosyaları düzenledi.
    Mümin organized the files.
    Mümin dosyaları deldi mi?
    Did Mümin punch the files?
    Mümin dosyaları deldi.
    Mümin punched the files.
11. Nihal meyve suyu yaptı.
    Nihal made juice.
    Nihal üzüm sıktı mı?
    Did Nihal squeeze grapes?
    Nihal üzüm sıktı.
    Nihal squeezed grapes.
12. Kedi bahçede oynadı.
    The cat played in the garden.
    Kedi çukur kazdı mı?
    Did the cat dig a hole?
    Kedi çukur kazdı.
    The cat dug a hole.
13. Sema gazete okudu.
    Sema read the newspaper.
    Sema bulmacayı çözdü mü?
    Did Sema solve the puzzle?

Sema bulmacayı çözdü.
Sema solved the puzzle.

14. Şevval başbakanla ilgili yazdı.
Şevval wrote about the prime minister.
Şevval başbakanı övdü mü?
Did Şevval praise the prime minister?
Şevval başbakanı övdü.
Şevval praised the prime minister.
15. Samet fare ile karşılaştı.
Samet encountered a mouse.
Samet fareden ürktü mü?
Did Samet get scared of the mouse?
Samet fareden ürktü.
Samet got scared of the mouse.
16. Semra yün kazaklar ördü.
Semra knitted wool sweaters.
Semra kazakları sattı mı?
Did Semra sell the sweaters?
Semra kazakları sattı.
Semra sold the sweaters.
17. Şoför ilerideki çevirmeyi gördü.
The driver saw the checkpoint ahead.
Şoför çevirmeden kaçtı mı?
Did the driver avoid the checkpoint?
Şoför çevirmeden kaçtı.
The driver avoided the checkpoint.
18. Yılmaz baş ağrısından yakındı.
Yılmaz complained of a headache.
Yılmaz hap yuttu mu?
Did Yılmaz swallow a pill?
Yılmaz hap yuttu.
Yılmaz swallowed a pill.
19. Araç muayeneye girdi.
The vehicle went for inspection.
Araç muayeneden geçti mi?
Did the vehicle pass inspection?
Araç muayeneden geçti.
The vehicle passed inspection.
20. Ekip kaygan yolu kontrol etti.
The team checked the slippery road.
Ekip tuz saçtı mı?
Did the team spread salt?
Ekip tuz saçtı.
The team spread salt.
21. Kamyon yağmurda ilerledi.
The truck moved in the rain.
Kamyon yolda kaydı mı?
Did the truck slip on the road?
Kamyon yolda kaydı.
The truck slipped on the road.
22. Tülay dedikoduları öğrendi.
Tülay learned the gossip.
Tülay dedikoduları yaydı mı?
Did Tülay spread the gossip?
Tülay dedikoduları yaydı.
Tülay spread the gossip.

23. Köpek koltuk üstünde oynadı.
    The dog played on the couch.
    Köpek koltuğu yoldu mu?
    Did the dog tear the couch?
    Köpek koltuğu yoldu.
    The dog tore the couch.
24. Bakan Doğu'ya ziyarette bulundu.
    The minister visited the east.
    Bakan Kars'a uçtu mu?
    Did the minister fly to Kars?
    Bakan Kars'a uçtu.
    The minister flew to Kars.
25. Feride İstanbul'a gitti.
    Feride went to Istanbul.
    Feride boğazda yüzdü mü?
    Did Feride swim in the Bosphorus?
    Feride boğazda yüzdü.
    Feride swam in the Bosphorus.
26. Tolga pastayı süsledi.
    Tolga decorated the cake.
    Tolga şam fıstığı ezdi mi?
    Did Tolga crush pistachios?
    Tolga şam fıstığı ezdi.
    Tolga crushed pistachios.
27. Vedat arabayı park etti.
    Vedat parked the car.
    Vedat dubaya çarptı mı?
    Did Vedat hit the buoy?
    Vedat dubaya çarptı.
    Vedat hit the buoy.
28. Berrin bostana gitti.
    Berrin went to the field.
    Berrin bostanı bozdu mu?
    Did Berrin ruin the field?
    Berrin bostanı bozdu.
    Berrin ruined the field.
29. Tamer demiri geçirdi.
    Tamer threaded the iron.
    Tamer demiri büktü mü?
    Did Tamer bend the iron?
    Tamer demiri büktü.
    Tamer bent the iron.
30. Faruk Norveç'e taşındı.
    Faruk moved to Norway.
    Faruk soğuktan bezdi mi?
    Did Faruk get fed up with the cold?
    Faruk soğuktan bezdi.
    Faruk got fed up with the cold.

Movie S1 (separate file). An example video showing the backward hand flip (3.01 s). Used with permission from info@turkeyvideonetwork.com. The backward hand flip is culture-specific and displays morpheme-like behavior in the grammatical encoding of negation in Turkish.
